# Lateral Approach in Robotic-Assisted Radical Prostatectomy: Introducing Gaston's Technique in Brazil

**DOI:** 10.1590/S1677-5538.IBJU.2025.0138

**Published:** 2025-04-15

**Authors:** Plinio Ramos Pinto, Thiago Camelo Mourão, Jayme Quirino Caon Nobre, Rodrigo Coelho de Carvalho, João Pedro Soares Nunes, Walter Henriques da Costa, Richard Pierre Gaston, Stenio Zequi

**Affiliations:** 1 AC Camargo Cancer Center Departamento de Urologia São Paulo SP Brasil Departamento de Urologia, AC Camargo Cancer Center, São Paulo, SP, Brasil; 2 Portuguesa de São Paulo Departamento de Uro-Oncologia Beneficência São Paulo SP Brasil Departamento de Uro-Oncologia Beneficência Portuguesa de São Paulo. São Paulo, SP, Brasil; 3 Clinique Saint Augustin Department of Urology Bordeaux France Department of Urology, Clinique Saint Augustin, Bordeaux, Nouvelle-Aquitaine, France

## Abstract

**Introduction:**

Prostate cancer is one of the most common malignancies in men, significantly impacting quality of life and survival (1, 2). Radical prostatectomy remains a key treatment for localized disease, with ongoing advancements in surgical techniques (3-5). The lateral approach in robotic-assisted prostatectomy was developed by Professor Richard Gaston and has emerged as a method designed to enhance anatomical preservation and functional outcomes, aligning with the growing demand for precision in prostate cancer management (6-8).

**Objective:**

To present for the first time in Brazil the step-by-step technique and initial experience with the lateral approach to radical prostatectomy, emphasizing its safety, feasibility, and reproducibility as a novel surgical option for prostate cancer treatment.

**Materials and Methods:**

This video demonstrates a lateral approach to radical prostatectomy in a 51-year-old male patient diagnosed with localized prostate cancer (Gleason 7 (3+^4^) in 2 out of 17 fragments). The surgical procedure was performed using a transperitoneal robotic approach, with lateral entry via the right paravesical space to optimize access and exposure of pelvic structures. Key technical steps included precise dissection of the endopelvic fascia, early identification and preservation of neurovascular bundles, and bladder neck preservation to enhance postoperative functional outcomes. Hemostasis was achieved using selective bipolar energy and clips, and urethrovesical anastomosis was performed using a running suture technique with barbed sutures.

**Results:**

The surgery was performed without complications, with an operative time of 150 minutes and estimated blood loss of 100 mL. The patient was discharged on the first postoperative day with adequate pain control. The urinary catheter was removed on the seventh postoperative day, and the patient reported complete continence from catheter removal onwards, requiring no pads. At three-month follow-up, the patient continued to report full urinary continence and satisfactory erectile function with phosphodiesterase type 5 inhibitors. His PSA levels remained undetectable at 3 and 6 months postoperatively.

**Conclusions:**

The lateral approach to radical prostatectomy represents a safe and reproducible technique for localized prostate cancer treatment. To our knowledge, this is the first reported case of this approach performed in Brazil, marking an important step in expanding surgical options for prostate cancer. Further studies are required to evaluate long-term clinical outcomes and comparative benefits.

Available at: http://www.intbrazjurol.com.br/video-section/20250138_Zequi_et_al
